# A Comparative Metabolomic Analysis Reveals Difference Manufacture Suitability in “Yinghong 9” and “Huangyu” Teas (*Camellia sinensis*)

**DOI:** 10.3389/fpls.2021.767724

**Published:** 2021-12-14

**Authors:** Xin Mei, Chuyuan Lin, Shihua Wan, Baoyi Chen, Hualing Wu, Lingyun Zhang

**Affiliations:** ^1^College of Horticulture, South China Agricultural University, Guangzhou, China; ^2^College of Biological Science and Agriculture, Qiannan Normal University for Nationalities, Duyun, China; ^3^Tea Research Institute, Guangdong Academy of Agricultural Sciences, Guangdong Provincial Key Laboratory of Tea Plant Resources Innovation and Utilization, Guangzhou, China

**Keywords:** *Camellia sinensis*, yellow-leaf mutant, metabolites analysis, flavonol glycosides, carotenoids, aroma compounds

## Abstract

“Yinghong 9” is a widely cultivated large-leaf variety in South China, and the black tea made from it has a high aroma and strong sweet flavor. “Huangyu” is a light-sensitive tea variety with yellow leaves. It was cultivated from the bud-mutation of “Yinghong 9” and has a very low level of chlorophyll during young shoot development. Due to chlorophyll being involved in carbon fixation and assimilation, the changes in photosynthesis might potentially affect the accumulation of flavor metabolites, as well as the quality of “Huangyu” tea. Although “Huangyu” has a golden yellow color and high amino acid content, the mechanism underlying the formation of leaf color and drinking value remains unclear. The widely targeted metabolomics and GC-MS analysis were performed to reveal the differences of key metabolites in fresh and fermented leaves between “Yinghong 9” and “Huangyu.” The results showed that tea polyphenols, total chlorophyll, and carotenoids were more abundant in “Yinghong 9.” Targeted metabolomics analysis indicated that kaempferol-3-glycoside was more abundant in “Yinghong 9,” while “Huangyu” had a higher ratio of kaempferol-3-glucoside to kaempferol-3-galactoside. Compared with “Yinghong 9” fresh leaves, the contents of zeaxanthin and zeaxanthin palmitate were significantly higher in “Huangyu.” The contents of α-farnesene, β-cyclocitral, nerolidol, and *trans-*geranylacetone, which were from carotenoid degradation and involved in flowery-fruity-like flavor in “Huangyu” fermented leaves, were higher than those of “Yinghong 9.” Our results indicated that “Huangyu” was suitable for manufacturing non-fermented tea because of its yellow leaf and flowery-fruity-like compounds from carotenoid degradation.

## Introduction

Tea (*Camellia sinensis*) is an important economic crop in south China. The sensory quality of tea products depends on their chemical compounds, although it is still challenging to illustrate. Color and flavor are two important sensory quality indicators for tea products. The leaf colors of tea cultivars often have severely effects on appearance and flavor which involved in manufacturing suitability of tea leaf (Zhang et al., 2017; Cheng et al., 2019; Hao et al., 2020). Many developers believe that the high-chlorophyll tea leaf is suitable for making green tea, while the low-chlorophyll leaf is suitable for making black or oolong tea. The main reason is that the low-chlorophyll tea leaf more easily turns yellowish or reddish during black tea or oolong tea processing. Recently, some white and yellow nature mutation varieties were cultured because of its yellow-green appearance of finished tea. Admittedly, yellow or white leaf tea mutations have high contents of amino acids, accompanied by low contents of tea polyphenols and total chlorophylls, which contribute to the qualities of white and yellow leaf tea (Feng et al., 2014). The discovery and revelation of the mechanism of leaf color mutants is a hot topic in tea plant breeding.

Previous studies showed that abnormal chlorophyll metabolism in nature mutation tea led to white or yellow bud and leaf. Many researches have been conducted on leaf color formation mechanisms in albino-cultivar during young shoot development using transcriptomics, metabolomics, and proteomics analysis (Li et al., 2011; Wang et al., 2013; Liu et al., 2017; Lu et al., 2019). Some research results showed that the yellow degree of leaves had a close relationship with the content of amino acids (Feng et al., 2014; Song et al., 2017). In nature, some natural bud mutants have been found to cope with the complex environment by changing their leaf color; this potential capability has been found in almost all higher plants (Huot et al., 2014; Li et al., 2020).

“Yinghong 9” (*Camellia sinensis var.* Yinghong No. 9) is a large leaf tea cultivar selected from Yunnandaye tea, it is a suitable cultivar for the manufacture of black tea because of its higher tea polyphenol content and flowery-aroma flavor. “Huangyu” is a yellow leaf mutant that bred from bud mutation of “Yinghong 9,” and it has a relatively low level of chlorophyll than the parent plant (Ma et al., 2016; Cheng et al., 2019). The changes of photosynthesis and metabolite accumulation in “Huangyu” might potentially affect the quality of the finished tea (Ma et al., 2016). So, it is interesting for the researchers to study the chemical compounds, differences, and manufacture suitability of “Yinghong 9” and “Huangyu” with the same genetic background.

Carotenoids and chlorophyll are the dominant pigments involved in the formation of leaf color of plants (Clément et al., 2015). The content of total chlorophylls and carotenoids, and especially their ratio (carotenoid/chlorophyll) in leaf, affect the environmental adaptability of plants during their development (Solovchenko, 2010; Gitelson, 2020). Long’s (Long et al., 2006) research showed that although carotenoid biosynthesis pathway would be changed in transgenic lines of tomato, which did not generally alter the content of phenolic or flavonoids. On the contrary, low chlorophyll and high carotenoids lead to a lower flavonoid in albino and yellow leaf tea, e.g., Anji baicha and Huangjinya. Some literature have shown that carotenoids were involved in the formation of aroma quality during tea processing. The products of carotenoid degradation, such as β-ionone, geranylacetone, β-cyclocitral, nerolidol and β-damascenone, are important aroma compounds of tea (Sanderson and Grahamm, 1973; Kawakami, 2002; Hazarika and Mahanta, 2010; Ho et al., 2015).

Currently, many aspects of coloration of the leaf and its influence on tea products and aroma formation are still poorly understood. “Huangyu,” a natural mutant variety of golden-yellow leaf tea, has a higher carotenoid content compared to its parent, with the same genetic background, offers us a chance on revealing the relationship of aroma quality with the yellow leaf color in tea. The purpose of our study is to elucidate whether the golden-yellow-leaf tea variety “Huangyu” has a better quality on the basis of its higher carotenoids and carotenoid-derived aroma compounds. This study also provides new insights into the mechanisms of the difference of quality and the manufacture suitability of “Huangyu” and “Yinghong 9.” The results of our study can provide theoretical evidence for tea breeding and utilization of unique wild tea resources.

## Materials and Methods

### Plant Materials and Sample Preparation

One bud and two leaves of *Camellia sinensis* cv. “Yinghong 9” were collected from the experimental tea garden of Tea Research Institute, Guangdong Academy of Agricultural Sciences (Yingde City, Guangdong Province, China) in August 2020. The mutant materials (“Huangyu”) were collected from the mutant plant, which was grafted on the parent plant (“Yinghong 9”). The tea leaves were equally divided into three portions. The samples were prepared by black tea processing described as follows: the fresh tea leaves were subjected to indoor natural withering at 25–27°C by spreading on bamboo sieves for 15 h. After withering, the leaves were rolled by a mini rolling machine for 60 min. Then the leaves were put into a bamboo-basket to ferment for 6 h at 25–27°C with a relative humidity of about 85%. During the black tea processing, fresh and fermented leaves were collected and immediately fixed with liquid nitrogen. And then the samples were ground to powder and stored in a freezer at −80°C before the following analysis. The water content of these leaves was measured at different stages during the black tea processing.

### Determination of Tea Polyphenol, Chlorophyll, and Carotenoids

#### Content Determination of Tea Polyphenols

The tea polyphenols content was determined by spectrophotometer method with minor modifications (Liang et al., 2003). The absorbance values at 540 nm were determined by a spectrophotometer (UNICO Instrument Co., Ltd., Shanghai, China). A control solution (5 ml distilled water, 5 ml reagent and 15 ml phosphate buffer) was determined on the same step. The calculation formula is as follows: tea polyphenols concentration (%) = (A × 1.957 × 2)/100 × L1/(L2 × M × m). A represents absorbance values. L1 represents the total volume of extracting solution, in ml. L2 represents the tested volume of extracting solution. M represents the dry weight of the sample, in g. The “m” represents dry-weight percentage of the sample in %.

#### Determination of Total Chlorophyll and Carotenoids

The determination of total chlorophyll and carotenoids contents were extracted and determined as previously described with some modifications (Song et al., 2017; Tian et al., 2019); The ground samples (500 mg) were extracted by 25 ml 95% ethanol (v/v) in darkness for 24 h at room temperature. After that, the extract was filtered and brought up to 25 ml using 95% ethanol (v/v), and then added 300 μl extracting into a 96 well plate, the absorbance values were determined using an automatic microplate reader (Sunrise, TECAN, Switzerland), and test wavelength is 663, 645, and 470 nm, respectively. The blank control used was a 95% ethanol (v/v). The content calculation equations of chlorophyll a, chlorophyll b, and carotenoids were as follows:


$$\mathrm{Chlorophyll}\text{a}=(12.21\times A_{663}-2.81\times A_{646})/\left(1000\times W\right)\times L;$$

Chlorophyllb=(20.13×A646−5.03×A)663/(1000×W)×L;

$$\text{Carotenoids}=(1,000\times A_{470}-3.27\times C_{\text{chlorophylla}}-104\times C_{\text{chlorophyll b}})/(229\times 1000\times W)\times L.$$

where A663, A646, and A470 represent the absorbance at 663, 646, and 470 nm, respectively; where V represents the total volume of extracting solution (ml), and W represents the fresh weight (FW) of the samples (g); The contents of chlorophyll and carotenoids were estimated in mg/g (FW).

### Metabolite Compounds Analysis in “Yinghong 9” and “Huangyu”

All above frozen samples were freeze-dried by LGJ-10 experimental vacuum freeze dryer (Songyuan Huaxing Technology Development Co., Ltd., Beijing, China). The freeze-dried samples were ground using a MM 400 grinder (Retsch, VERDER Group, Germany) with a zirconia bead for 90 s at 30 Hz. The lyophilized sample (100 mg) was extracted with 1.2 mL 70% methanol solution for 24 h at 4°C. During this period, the samples were vortexed (vortex-genie2, Scientific Industries, United States) 30 s once every 30 minutes for a total of six times to facilitate the extraction. Following centrifugation at 12,000 *g* for 15 min, the supernatant was filtered with SCAA-104 (0.22 μm microporous filter membrane, ANPEL, Shanghai, China) and analyzed by UPLC-MS/MS.

The samples were analyzed using Ultra Performance Liquid Chromatography Tandem Mass Spectrometry (UPLC-MS/MS) according to the procedure described by Zhou C. et al. (2020). The chromatographic separations were conducted on an Agilent UPLC column SB-C18 (1.8 μm, 2.1 mm × 100 mm) at 40°C. The mobile phase consisted of solvent A, pure water with 0.1% formic acid, and solvent B, acetonitrile with 0.1% formic acid. Sample measurements were performed with a gradient program that employed the starting conditions of 95% A, 5% B. Within 9 min, a linear gradient to 5% A, 95% B was programmed, and a composition of 5% A, 95% B was kept for 1 min. Subsequently, a composition of 95% A, 5% B was adjusted within 1.10 min and kept for 2.9 min. The flow rate of the mobile phase was 0.35 ml/min; The injection volume was 4 μl. The metabolites were identified by using the primary and secondary spectral data annotated on public databases (MassBank^1^) and proprietary database (Metware Database, Metware Biotechnology Co., Ltd., Wuhan, China) following the previously operating procedures (Rothenberg et al., 2019). Metabolite quantification was conducted using MRM. In order to monitor the stabilization of analyzing equipment, two mixture samples were run for quality control after every 10 samples processing. Unsupervised PCA (principal component analysis) was performed by statistics function prcomp within R^2^. Significantly differential metabolites between groups were determined when its variable importance in the project (VIP) ≥ 1 and fold change ≥ 2 or ≤ 0.5. VIP values were extracted from orthogonal partial least squares discriminant analysis (OPLS-DA) results, which were conducted using R package (Chong and Xia, 2018).

### Isolation and Analysis of Carotenoid Compounds

The freeze-dried samples were ground (30 Hz, 1 min) using a MM 400 grinder (Retsch, VERDER Group, Germany). Fifty milligrams of ground samples were mixed with an appropriate volume of the extraction solution (ethanol: acetone: n-hexane, 2:1:1,v/v/v) with 0.01% butylated hydroxytoluene (g/mL) and internal standard. The mixture was adequately vortexed (Jingmei, Shanghai, China) at room temperature for 20 min. The mixture solution was then centrifuged (5424R, Eppendorf, Germany), and the supernatant was collected. The samples were repeatedly extracted two times using the same method, and the supernatants were combined and concentrated. The concentration of extracts redissolved in mixture solution (methanol: methyl tertiary-butyl ether, 3:1, v/v). The solutions were filtrated by SCAA-104 filter (0.22 μm, Anpel laboratory technologies Inc., Shanghai, China) before UPLC-MS/MS analysis. Carotenoid identification and quantification analysis were performed by UPLC-MS/MS system at Metware Biotechnology Company (Wuhan, China). UHPLC Chromatographic and APCI-Q Trap-MS/MS conditions were conducted following the previously stated procedure (Yan et al., 2019). Carotenoids were quantitatively analyzed as described in Zhou W. et al. (2020).

### Volatile Compounds Determination by GC–MS

Volatile constituents were extracted according to the previous study (Mu et al., 2018; Shi, 2018) with minor changes. Briefly, ground tea powder (1.0 g) was accurately weighed into a homemade extraction bottle (50 mL), and 3 ml water and 1 50 μl decanoic acid ethyl ester (100 μM, internal standard) was added. The extraction bottle was placed in an air bath (45°C) to equilibrate for 5 min, volatile constituent was extracted using a headspace solid-phase microextraction (HS-SPME) device (57310U, 65 μm PDMS/DVB, Supelco, Bellefonte, PA, United States) in a water bath (45°C) for 37 min. After extraction, the SPME fiber was immediately inserted into the GC injector for desorption at 250°C for 5.5 min. Volatile constituents were analyzed using GC–MS (Agilent GC 6890N-5973, Agilent, Santa Clara, CA, United States) as previously described (Mu et al., 2018). GC/MSD MassHunter workstation software (Agilent, Santa Clara, CA, United States) was used for GC–MS data analyses including peaks extracting, data baselines filtering, calibration of the baseline, peak alignment, deconvolution analysis, and integration of the peak area. Identification was performed by comparing mass spectra with NIST14.L libraries databases. To compare the differences of “Huangyu” and “Yinghong 9,” the critical volatiles was selected by retention time as an approximate guide (Wu et al., 2019).

### Determination of Enzyme Activities

The carotenoid cleavage crude enzyme extracts were carried out as a method described before (Ningrum and Schreiner, 2014). The polyphenol oxidase enzyme extracts were carried out as follows: 1,000 mg frozen samples were weighed into the mortar, add 5 mL precooling phosphate buffer (pH 7.0) with 300 mg polyvinylpolypyrrolidone (PVPP), and the mixture was sufficiently ground on the icebox. Then the mixture was washed into a 20 ml centrifugal tube using 5 ml buffer. The homogenate was centrifuged at 15,000 *g* for 20 min at 4°C, and then the supernatant was immediately used for enzyme assays. The enzyme activities of samples were determined according to the protocol of the assay kits, respectively (Polyphenol Oxidase assay kit, Suzhou Keming Biotechnology Co., Ltd. β-Glucosidase assay kit and Plant Carotenoid Cleavage Dioxygenase Elisa kit, Beijing Solarbio Science & Technology Co., Ltd.).

### Data Analysis

Statistical analysis was carried out with SPSS software (IBM version 24.0 for Windows, SPSS Inc., Chicago, IL, United States). Significant differences between different groups were determined using Tukey’s *post hoc* test. *P* value less than 0.05 was considered statistically significant. Excel 2010 (Microsoft, United States) was applied to drawbar graphs of the experimental data. Flavonol metabolites heat map was prepared using pheatmap in R package (**??**). The carotenoids heat map was prepared on major bio cloud platform (Major Biotechnology Co., Shanghai, China^3^).

## Results

### Pigments Content in “Yinghong 9” and “Huangyu”

**FIGURE 1:**
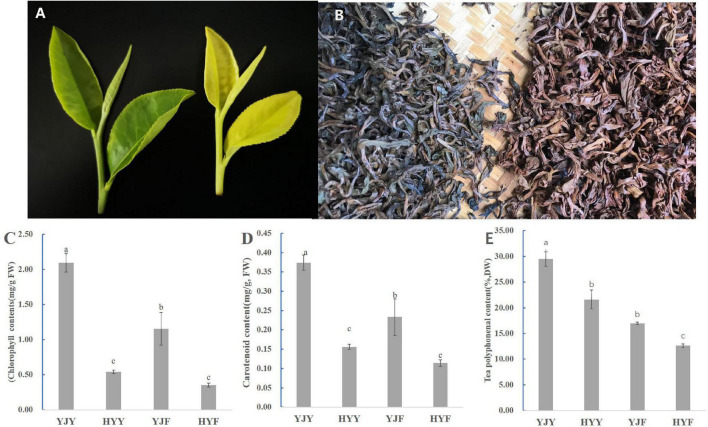
Phenotypes and pigment content in “Yinghong 9” and “Huangyu” leaves. **(A)** Leaf phenotypes of “Yinghong 9” and “Huangyu.” **(B)** Appearance of “Yinghong 9” and “Huangyu” fermented leaves. **(C)** Total chlorophyll content of “Yinghong 9” and “Huangyu” leaves. **(D)** Carotenoid content of “Yinghong 9” and “Huangyu” leaves. **(E)** Total tea polyphenols content of “Yinghong 9” and “Huangyu” leaves. The data are presented as the mean standard deviation (*n* = 3). Means with different letters at each treatment represent a significant difference at *p* ≤ 0.05. YJY was “Yinghong 9” fresh leaves, YJF was “Yinghong 9” fermented leaves; HYY was “Huangyu” fresh leaves, HYF was “Huangyu” fermented leaves.

The leaves of “Yinghong 9” show normal green leaf and bud, while its mutant “Huangyu” has yellow leaf and bud (Figure 1A). The fermented leaves from “Huangyu” were bright red compared with “Yinghong 9” of which leaves show dark brown color (Figure 1B). The leaf color of “Yinghong 9” and “Huangyu” was affected by pigment composition and proportion, including the contents of tea polyphenols, chlorophyll a, chlorophyll b, carotenoids, and their ratio. The current results showed that the contents of tea polyphenols and total chlorophyll in “Yinghong 9” leaves were higher than that of “Huangyu,” In contrast, the content of carotenoids in “Huangyu” leaves was still lower than those in normal green leaf parent, “Yinghong 9” (Figures 1C,D). The contents of all three pigments in “Yinghong 9” and “Huangyu” fermented leaves were significantly lower (*p* < 0.05) than those in fresh leaves (Figures 1C–E).

### Differential Metabolites in “Yinghong 9” and “Huangyu”

**FIGURE 2:**
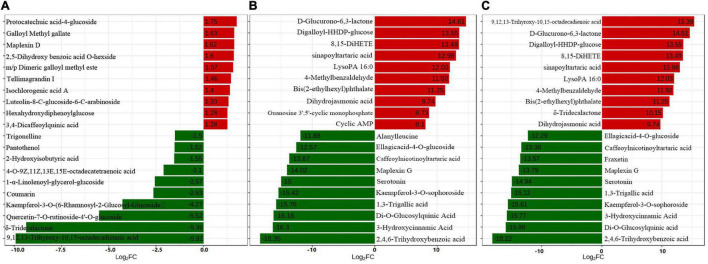
The top 20 differential metabolites between “Yinghong 9” (YJ) and “Huangyu” (HY). **(A)** Top 20 FC differential metabolites between “Yinghong 9” (YJ) and “Huangyu” (HY), fold change (YJY/HYY). **(B)** Top 20 FC differential metabolites between fresh leaves (YJY) and fermented leaves (YJF)of “Yinghong 9,” fold change (YJY/YJF). **(C)** Top 20 FC change metabolites between Fresh leaves (HYY) and Fermented leaves (HYF)of “Huangyu,” fold change (HYY/HYF).

A total of 609 metabolites were identified in “Yinghong 9” and “Huangyu” fresh and fermented leaves by UPLC-MS/MS analysis, and representative differential metabolites, including 26 up-regulated and 20 down-regulated metabolites were identified in “Huangyu” and “Yinghong 9” fresh leaves (fold change (FC) ≥ 2 or ≤ 0.5) (Supplementary Table 1). Interestingly, among the top 20 down-regulated metabolites, 9,12,13-Trihyroxy-10,15-octadecadienoic acid and δ-tridecalactone showed markedly different in “Yinghong 9” and “Huangyu” fresh leaves (Figure 2A). The reason might be that the two compounds were almost undetectable in “Huangyu.” Among the 26 up-regulated metabolites, protocatechuic acid-4-glucoside showed the highest increased level in “Huangyu” fresh leaves, increasing about 3.37-fold (Figure 2B). It was noteworthy that most of the top 10 up-regulated metabolites were phenolic compounds (phenolic acids or tannins). In comparison, most of the top 10 down-regulated metabolites were lipids (high content in “Yinghong 9”) (Figure 2C). The difference was possibly due to metabolic flux in the two cultivars because of their different leaf color.

Compared with “Yinghong 9” fresh leaves, there were 408 differential metabolites in fermented leaves (225 up-regulated and 183 down-regulated), while 439 differential metabolites in “Huangyu” fresh leaves were different from fermented leaves (255 up-regulated and 184 down-regulated) (Supplementary Tables 2, 3). The results show that the highest increase in up-regulated metabolites was D-glucurono-6,3-lactone, while the most significant change in down-regulated metabolites was glutathione during “Yinghong 9” fermented processing. Most of them were organic acids or phenolic acids, which implied that organic acids or phenolic acids were converted to new metabolites during black tea fermentation. The top ten up-regulated and down-regulated metabolites during “Yinghong 9” fermented processing were very similar.

### Comparison of Differences in Flavonol Glycosides in “Yinghong 9” and “Huangyu”

**FIGURE 3:**
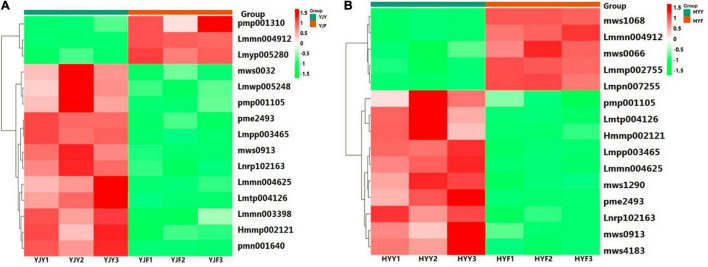
Heat map of differential flavonol glycosides clusters in “Yinghong 9” and “Huangyu.” **(A)** Top 15 FC change flavonol glycosides between fresh leaves (YJY) and fermented leaves (YJF)of “Yinghong 9.” **(B)** Top 15 FC change flavonol glycosides between fresh leaves (YJY) and fermented leaves (YJF) of “Huangyu.” In the heatmap, differential flavonol glycosides marked with green and red were down- and upregulated in “Yinghong 9” or “Huangyu,” respectively. pmp001310,6-Hydroxykaempferol-3,6-O-Diglucoside; Lmmn004912,Quercetin-3-O-methyl ether; Lmyp005280, Isorhamnetin-3-O-[6″-p-coumarylglucosyl-O-rhamnoside; Lmmn003398, Kaempferol-3-O-(6″-acetyl)glucoside; Lmmn004625, Dihydrokaempferol-7-O-glucoside; Lmwp005248, Kaempferol-3-O-(6”’-p-Coumaroylglucosyl)-Glucoside-7-Rhamnoside; mws0913, Kaempferol-3-O-galactoside; mws0032, Myricetin; pmp001105, Kaempferol-3-O-neohesperidoside-7-O-glucoside; pme2493, Kaempferol-3,7-O-dirhamnoside; Lnrp102163, Quercetin-3-O-rutinoside-7-O-rhamnoside; Hmmp002121, Isorhamnetin-3-O-gallate Lmpp003465, Myricetin-3-O-glucoside; Lmtp004126, Myricetin-3-O-(6″-malony)glucoside; pmn001640, Myricetin-3-O-arabinoside. Lmmp002755, Quercetin-7-O-rutinoside-4’-O-glucoside; Lmpn007255, 6-Methoxyquercetin; mws0066, Isorhamnetin; mws1068, Kaempferol; mws1290, Kaempferol-3-O-(6″-p-coumaroyl)glucoside; mws4183, Quercetin-3-*O*-α-L-arabinopyranoside.

Altogether, 20 flavonol glycosides were identified in the “Yinghong 9” and “Huangyu” leaves as shown in Supplementary Table 4, including eight kaempferol-3-glycosides, ten quercetin-3-glycosides, and two myricetin-3-glycosides. We observed that the content of flavonol glycosides was slightly different in “Yinghong 9” and “Huangyu” fresh leaves, but the changes of flavonol glycosides were different during the two cultivars fermentation. Quercetin-3-*O*-xyloside was the most abundant detected compound in “Yinghong 9” and “Huangyu” fermented leaves (Supplementary Table 4). But the highest content of the top 15 changes was myricetin-3-*O*-arabinoside in “Yinghong 9” fermented leaves (Figure 3A and Supplementary Table 5A), while it was quercetin-3-*O*-α-L-arabinopyranoside in “Huangyu” fermented leaves (Figure 3B and Supplementary Table 5B). In addition, the content of kaempferol-3-glucoside, kaempferol-3-*O*-galactoside and kaempferol-3-glucoside to kaempferol-3-galactoside ratio were significantly different. The content of kaempferol-3-glucoside in “Yinghong 9” was 1.5 fold more than that of “Huangyu” (Supplementary Table 4). The significant difference in kaempferol-3-glucoside and its ratio to kaempferol-3-galactoside indicates the potential for the discrimination of the manufacture suitability of tea varieties.

### Comparison of Carotenoids Metabolites in “Yinghong 6” and “Huangyu”

**FIGURE 4:**
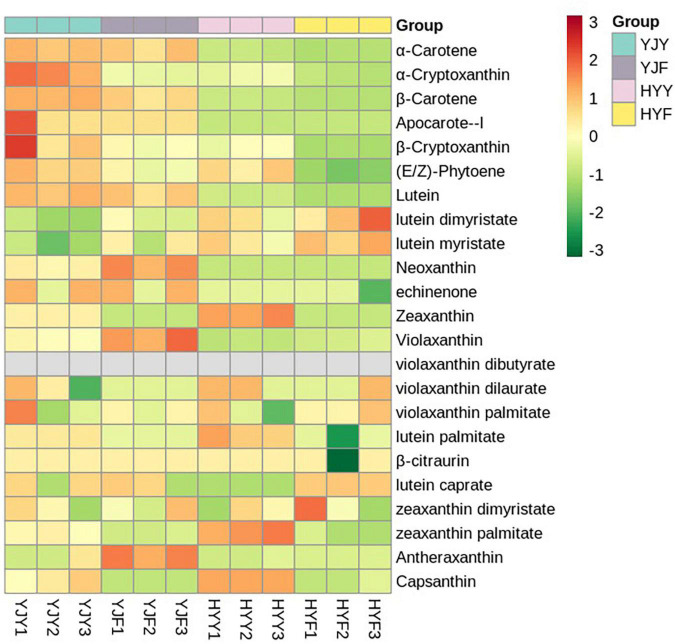
Heat map of volatile compounds clusters in “Yinghong 9” and “Huangyu” fresh and their fermented leaves. Red and green indicate higher and lower abundances, respectively. YJY was “Yinghong 9” fresh leaves, YJF was “Yinghong 9” fermented leaves; HYY was “Huangyu” fresh leaves, HYF was “Huangyu” fermented leaves.

A total of 23 carotenoids were identified in “Yinghong 9” and “Huangyu” fresh leaves, including a high content of α-carotene, β-carotene, lutein; zeaxanthin, and minor compounds, such as antheraxanthin, violaxanthin, neoxanthin, apocarote, α-cryptoxanthin, β-cryptoxanthin, capsanthin, (E/Z)-phytoene, apocarotenal, zeaxanthin, lycopene, violaxanthin and neoxanthin (Supplementary Table 6). The types and concentrations of carotenoids were significantly different between “Yinghong 9” and “Huangyu.” Compared with “Huangyu” fresh leaves, the contents of lutein, α-carotene, (E/Z)-phytoene, β-cryptoxanthin, β-carotene, and neoxanthin were significantly higher in “Yinghong 9.” While the content of zeaxanthin was significantly higher in “Huangyu” leaves (Figure 4 and Supplementary Table 6).

Most of the carotenoids significantly decreased in “Yinghong 9” fermented and “Hongyun” fermented leaves, e.g., β-carotene, lutein, zeaxanthin. On the contrary, some carotenoids significantly increased in fermented leaves, such as antheraxanthin, violaxanthin, and neoxanthin in “Yinghong 9” fermented leaves; lutein caprate and violaxanthin in “Huangyu” fermented leaves. Interestingly, lutein caprate even was not detected in “Yinghong 9” fresh leaves, which markedly increased in “Huangyu” fermented leaves. The reason may be that the lutein caprate is the product of β-carotene degradation in fermented leaves.

### Volatile Compounds Profiling of “Yinghong 9” and “Huangyu” Leaves by SPME-GC–MS

**FIGURE 5:**
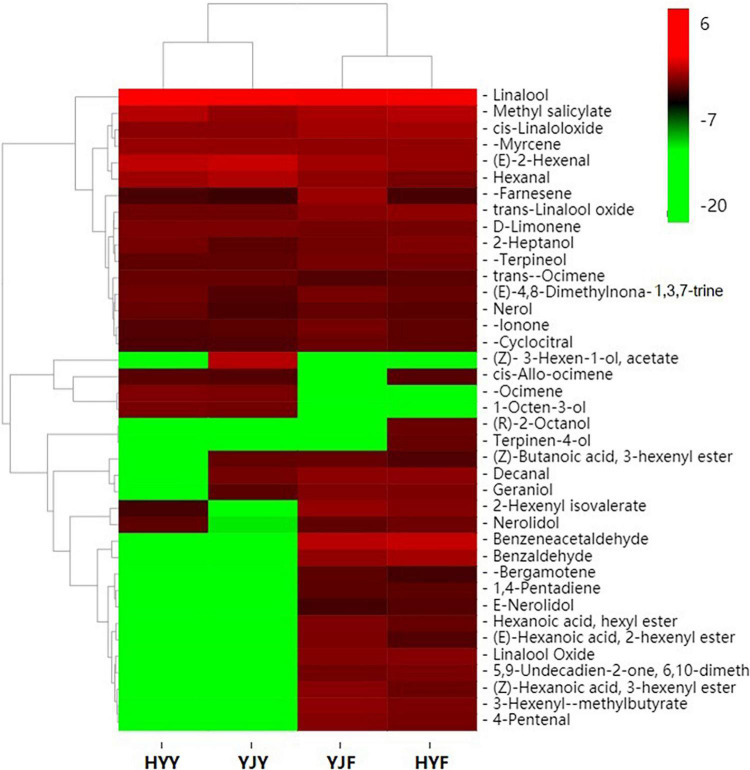
Heat map of carotenoids metabolite clusters in “Yinghong 9” and “Huangyu” fresh and their fermented leaves. Red and green indicate higher and lower abundances in respective. YJY was “Yinghong 9” fresh leaves, YJF was “Yinghong 9” fermented leaves; HYY was “Huangyu” fresh leaves, HYF was “Huangyu” fermented leaves.

In order to study the differences of volatile compounds, volatile compounds analyses were carried out in “Yinghong 9” and “Huangyu” fresh leaves and fermented leaves (Figure 5). Twenty-five volatile compounds were identified in “Yinghong 9” fresh leaves, while 21 volatile compounds were identified in “Huangyu” fresh leaves (Supplementary Table 7). Higher contents of hexanal, (E)-2-hexenal were detected in “Yinghong 9” – both of which were involved in conferring the grassy-green smell of fresh leaves or finished tea (Supplementary Table S7). Meanwhile, higher contents of 3-Hexen-1-ol, acetate, decanal and geraniol were detected in “Yinghong 9.” All of them were involved in the flowery-fruity-like flavor of finished tea. But correspondingly, the contents of linalool, methyl salicylate, nerolidol were higher in “Huangyu” than that of “Yinghong 9” fresh leaves.

As confirmed by previous research, new volatile compounds were generated as the fermentation process proceeds. In the present study, the numbers of volatile compounds increased from 22 (fresh leaves) to 35 (fermented leaves) in “Yinghong 9” (Supplementary Table 7). Similar increase took place in “Huangyu” cultivar, which was from 22 (fresh leaves) to 35 (fermented leaves). The content of linalool, 1-octen-3-ol, β-ocimene, *cis-*allo-ocimene and 3-Hexen-1-ol, acetate significantly decreased during the fermentation of “Yinghong 9” as well as “Hongyun” cultivars. Some new volatile compounds, e.g., benzaldehyde, benzeneacetaldehyde, linalool oxideI, linalool oxide II,3-hexenyl-α-methylbutyrate, 2-hexenyl isovalerate, geraniol, *cis-*3-hexenyl hexanoate, *cis-*hexenyl hexanoate, *cis-*2-hexenyl hexanoate, α-bergamotene, 6,10-dimethyl-5,9-undecadien-2-one (*trans-*geranylacetone) and nerolidol were detected in “Yinghong 9” and “Huangyu” fermented leaves. At the same time, the contents of α-farnesene, β-ionone, β-cyclocitral and nerolidol in fermented leaves were significantly higher than those of fresh leaves.

### Difference of Enzyme Activities in Fresh and Fermented Leaves in “Yinghong 9” and “Huangyu”

**FIGURE 6:**
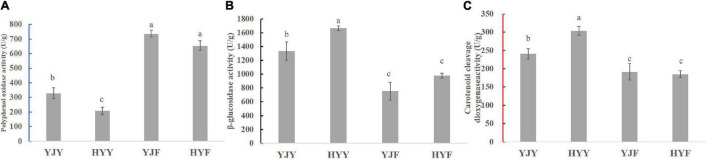
Comparison of enzyme activities in fresh and fermented leaves of “Yinghong 9” and “Huangyu.” **(A)** Polyphenol oxidase activity of “Yinghong 9” and “Huangyu” leaves. **(B)** β-glucosidase activity of “Yinghong 9” and “Huangyu” leaves. **(C)** Carotenoid cleavage dioxygenase activity of “Yinghong 9” and “Huangyu” leaves. All the data are presented as the mean standard deviation (*n* = 3). Means with different letters at each treatment represented a significant difference at *p* ≤ 0.05. YJY was “Yinghong 9” fresh leaves, YJF was “Yinghong 9” fermented leaves; HYY was “Huangyu” fresh leaves, HYF was “Huangyu” fermented leaves.

To reveal the manufacture suitability of “Yinghong 9” and “Huangyu,” the enzyme activity of polyphenol oxidase β-glucosidase and carotenoid cleavage dioxygenase were measured in their fresh leaves and fermented leaves. The results showed that the activities of polyphenol oxidase, β-glucosidase in “Yinghong 9” were higher than that of “Huangyu,” Similarly, the activity of carotenoid cleavage dioxygenase (CCD) showed also had the same change tendency in “Yinghong 9” and “Huangyu” fresh leaves (Figure 6). The difference was that the activity of polyphenol oxidase in fresh taste was higher than that of fermented leaves, but the activities of the other two enzymes, (β-glucosidase and carotenoid cleavage dioxygenase) decreased significantly in fermented leaves.

*B*-Glucosidase is one of the important enzymes involved in tea aroma compounds formation (Zeng et al., 2019), which can hydrolyze hydrolysis of β-1,4- glycosidic linkages and release free volatile compounds such as linalool, benzaldehyde, and methyl salicylate during tea processing (Yang, 2013). these compounds had flowery fruity fragrances and were beneficial to the aroma quality of black tea. As shown in Figure 6, the activity of the β-glucosidase in “Huangyu” was significantly higher than that of “Yinghong 9.” The activity of the β-glucosidase decreased significantly in “Yinghong 9” and “Huangyu” fermented leaves. The decrease in enzyme activity in the fermented stage might be due to the interaction between the enzyme and polyphenol compounds, as well as the decrease of substrate concentration (Supriyadi et al., 2021). So, after the withered tea leaf was subjected to rolling, the increase of tea polyphenols and substrates decreases led to a decrease in the activity of the β-glucosidase during fermentation processing. This mechanism has been verified by some research findings (Wang et al., 2001a,b).

**FIGURE 7:**
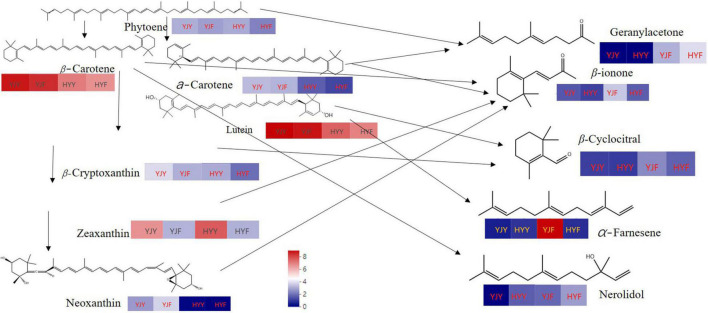
The content of main types of carotenoids metabolism schematic and degradation products (main aroma compounds). Red and blue indicate higher and lower abundances, respectively. YJY was “Yinghong 9” fresh leaves, YJF was “Yinghong 9” fermented leaves; HYY was “Huangyu” fresh leaves, HYF was “Huangyu” fermented leaves.

Wang et al. (2020) had confirmed that both carotenoid cleavage dioxygenase1 (CsCCD1) and carotenoid cleavage dioxygenase4 (CsCCD4) are involved in the formation of β-ionone during tea manufacture. In this study, the carotenoid cleavage dioxygenase activity was significantly higher in “Huangyu” than in “Yinghong 9” fresh leaves (Figure 6). That might be the reason why the content of aroma components was still high in “Huangyu” fresh leaves, although the total amount of carotenoids was lower than “Yinghong 9.” The results might be partially due to aroma compounds coming from glycoside hydrolysis and carotenoid degradation pathways (Figure 7). Now that there was a higher content of zeaxanthin and E-nerolidol, 6,10-dimethyl-5,9-undecadien-2-one, nerolidol in “Huangyu,” which were the degradation product of carotenoids, the degradation mechanism of carotenoids needs to be studied further. The results also suggested that “Huangyu” was still a variety which had good manufacture suitability. Furthermore, now that the decrease of carotenoid cleavage dioxygenase activity in fermented leaves indicates that the “Huangyu” variety is more suitable for manufacturing non-fermented tea.

## Discussion

### The Yellow Leaf Phenotype Is Closely Associated With Chlorophyll and Carotenoid Metabolism

Tea, produced from fresh tea (*Camellia sinensis*) leaves, is an important, widely popular non-alcohol beverage due to its health benefit functions, as well as its pleasant flavor. Unlike most other agricultural products, the color of infused leaves is involved in the sensory quality formation of tea. Thus, recently the researchers paid more attention to the study on the relationship of leaf color and characteristic compounds, and its influence on health benefits. In fact, compared to typical green leaves, several albino tea varieties have been successfully developed into new products because of their precious and tasteful quality, e.g., “Anji baicha,” “Yujingxiang,” and “Huangjinya.” Unluckily, tea breeding goals have been limited to a brighter color or better taste. Other valuable traits such as yield, flavor value, and manufacture suitability have long been ignored.

Previous studies indicated that the formation of tea leaf color from white to purple might be due to changes in different pigments and their ratios. In general, the greening of leaves was mainly due to the higher concentrations of chlorophylls, and the albino-induced white and yellow leaves were mainly due to lower concentrations of chlorophyll, while certain concentrations of carotenoids dominate in leaf formation (Zhu et al., 2015; Tsai et al., 2017). Similar research results had been proven in special light-sensitive albino yellow leaves cultivars such as “Yujinxiang,” “Huangjinya” (Song et al., 2017). The current study results showed that total chlorophyll was significantly higher contents in “Yinghong 9” normal green leaves than in the “Huangyu” yellow leaves (Figure 2A). The content of tea polyphenols and carotenoids changed in a manner similar to that observed for chlorophyll in two cultivars. The content of tea polyphenols, total chlorophyll, and carotenoid decreased significantly during the fermentation of “Yinghong 9” and “Huangyu” leaves.

As previously reported, carotenoids as an important pigment in green leaves could maintain efficient photosynthesis, by scavenging various reactive oxygen free radicals and protecting chlorophylls from photooxidation. The low chlorophyll content in yellow leaves was due to the blocked chlorophyll synthesis and enhanced degradation of intermediate products. It was speculated that the yellow leaves under the strong light of “Huangjinya” were due to the color presentation of carotenoids and flavonoids after chlorophyll content was significantly reduced. In this study, the types and contents of carotenoids were significant differences between “Yinghong 9” and “Huangyu.” Compared with “Huangyu” fresh leaves, the content of lutein, α-carotene, (E/Z)-phytoene, β-cryptoxanthin, β-carotene, and neoxanthin were significantly higher in “Yinghong 9.” In comparison, the content of zeaxanthin, and zeaxanthin palmitate were significantly higher in “Huangyu” leaves. Previous studies showed that the changes in the abundances of carotenoids were closely relevant to the expression of carotenoid biosynthesis genes (Zhang et al., 2012).

In plants, zeaxanthin is an essential metabolite of β-carotene. β-carotene generates zeaxanthin through the catalysis of β-carotene hydroxylase (BCH) (Kim et al., 2009). Silencing the BCH genes leads to an increase in the levels of β-carotene and carotenoids but reduces that of zeaxanthin in potato tubers (Diretto et al., 2007). Similarly, the expression levels of the β-arotene hydroxylase gene were up-regulated in yellow leaf wheat, which enhanced the metabolic flux from β-carotene to zeaxanthin, and resulted in increasing the content of zeaxanthin and decreasing the content of β-carotene (Wu et al., 2018). Similar results had been reported in light-sensitive tea “Huangjinya” and “Yujinxiang” cultivars (Liu et al., 2017). The difference was that the content of lutein, β-carotene and zeaxanthin was higher in “Huangjinya,” while zeaxanthin, carotene, violaxanthin, cryptoxanthin, and lutein content were higher in “Yujinxiang.” Interestingly, as downstream products of zeaxanthin, the content of violaxanthin, neoxanthin, and antheraxanthin was higher in “Yinghong 9” than in “Huangyu.” The difference could be due to the synthesis of the downstream product resulting in reducing substrate content. Altogether, despite the content of total carotenoid being higher in “Yinghong 9,” chlorophyll might conceal the coloration of carotenoids and result in the formation of green in “Yinghong 9” normal leaf. But for “Huangyu,” the absence of chlorophyll in leaf, leaf coloration is predominated by carotenoids.

### The Type and Content of Flavonol Glycosides in “Yinghong 9” and “Huangyu” Lead to Their Different Manufacture Suitability

Flavonol glycosides were one of the most important groups of polyphenols besides catechins in tea. Previous studies showed that flavonol glycosides were the critical ingredient of green tea soup, in which they color the tea liquor as yellow or yellow-green pigments (Dai et al., 2017). Like catechins, flavonol-glycosides play an important role in forming the tea taste (Scharbert et al., 2004; Scharbert and Hofmann, 2005). Not only can they engender dry and smooth-astringency sensations in the mouth, but also, they are involved in enhancing the bitterness of tea soup. The previous researchers confirmed that kaempferol-3-glucoside and kaempferol-3-galactoside were both astringent components, of which the taste threshold of kaempferol-3-glucoside and kaempferol-3-galactoside were 0.67 and 6.7 μmol/L, respectively (Scharbert et al., 2004; Scharbert and Hofmann, 2005). The difference of taste threshold of the two flavonol-glycosides was up to ten times. In contrast, the taste thresholds of quercetin-3-glucoside and quercetin-3-galactoside were 0.65 and 0.43 μmol/L, respectively, with a difference of 1.5 times. Therefore, the ratio of kaempferol-3- glucoside to kaempferol-3-galactoside had an important impact on tea taste.

Glucosylation and galactosylation were two competing glycosylation metabolic ways. Simultaneously the content of them varies among different tea varieties. The content of galactosylated flavonol in tea shoots leads to better manufacturing-suitability of green tea. In contrast, the content of glucosylated flavonol was significantly higher in black tea manufacturing-suitability varieties (Dai et al., 2017). This implies that the variety with more galactosylation of flavonol is suitable for manufacturing green tea and white tea (non-fermented tea), while the variety with more glucosylation of flavonol may be suitable for manufacturing black tea and oolong tea (fermented tea) (Jiang et al., 2015).

In this study, the content of kaempferol-3- glucoside in “Yinghong 9” was 1.5 times more than that of “Huangyu.” And the ratio kaempferol-3-glucoside to kaempferol-3-galactoside was lower, which indicates that the glycosylation metabolism of kaempferol in tea plants flowed toward galactosylation, which was beneficial for reducing the content of kaempferol-3-glucoside. So, “Huangyu” is suitable for manufacturing non-fermented tea, e.g., green tea and white tea. Correspondingly, “Yinghong 9” was suitable for manufacturing black tea since it had a high concentration of kaempferol-3-glucoside as well as a lower ratio of kaempferol-3-glucoside to kaempferol-3-galactoside (Supplementary Table 5). Liu et al. (2017) found that compared to the green variety, metabolic flux convert to flavonoid and carotenoids pathways to a certain extent in “Yujingxiang” yellow leaf variety. The change of metabolism flux might enhance the production of the antioxidant quercetin or quercetin glycosides rather than catechin biosynthesis. However, our present results were not consistent in this conclusion. It seemed that the yellow leaf “Huangyu” as a rare variety was worth further study because of its complex metabolic flux.

### The Differences of Glycosides Hydrolysis and Carotenoids Degradation Might Contribute to “Huangyu”xs Fresh Leaves Aroma Quality

Tea aroma quality, which is composed of the volatile compounds that originated during tea processing, is an important quality factor and determines dry tea’s economic benefit. A large number of literatures was concerned about analysis technology, non-carotenoid volatile compounds formation during tea processing, as well as the difference of varieties. The relationship between the carotenoids and their volatile components in yellow leaf tea varieties has received little attention. The formation of volatile compounds from carotenoids comes from the various degradation pathways. Evidence showed that β-ionone, a critical flowery component was the product of β-carotene oxidation degradation, in addition, linalool, nerolidol, β-cyclocitral and geranylacetone were degradation products of other carotenes present in tea (Ravichandran, 2002). In fact, as critical yellow pigments, carotenoids were present in nearly all green or yellow leaf tea, and their degradation promotes the formation of flowery aroma components during black tea processing (Hazarika and Mahanta, 2010). It is interesting to note that Ravichandran has reported that compared with Assam varieties, China varieties had more carotenoids in fresh leaves (Ravichandran, 2002), which implied that China varieties were better manufacture suitability for high aroma black tea. We really should pay our attention to China’s large leaf and yellow leaf mutant variety such as “Yinghong 9” and “Huangyu.”

As stated above, β-Glucosidase was involved in tea aroma compounds formation, which could hydrolyze hydrolysis of β-1,4- glycosidic linkages and release free volatile compounds such as linalool, benzaldehyde and methyl salicylate during tea processing (Yang, 2013). Our study results showed that the relatively high concentration of linalool, methyl salicylate, nerolidol was detected in “Huangyu” fresh leaves. Accordingly, the activity of the β-glucosidase in “Huangyu” was significantly higher than that of “Yinghong 9” (Figure 6B).

Similarly, the content of zeaxanthin, and zeaxanthin palmitate were significantly higher in “Huangyu” leaves, while the contents of nerolidol, α-farnesene and geranylacetone were significantly low in “Yinghong 9” fresh leaves, but the contents of these compounds were higher in ‘Huangyu’ fresh leaves. Correspondingly, the carotenoid cleavage dioxygenase activity was significantly higher in “Huangyu” than in “Yinghong 9” fresh leaves (Figure 6C). Due to all three of the compounds were carotenoids degradation products, it was reasonable to presume that the differences of carotenoids might influence aroma compounds in “Yinghong 9” and “Huangyu” fresh leaves. Despite some new volatile compounds such as benzaldehyde, benzeneacetaldehyde, linalool oxideI, linalool oxideII, and so on were detected in “Yinghong 9” and “Huangyu” fermented leaves. Particularly, the contents of α-farnesene, β-ionone, β-cyclocitral, and geranylacetone had a fruity floral odor and contribute significantly to black tea aroma quality were increased in “Yinghong 9” fermented leaves. However, the activities of β-glucosidase and carotenoid cleavage dioxygenase decreased greatly in “Huangyu” fermented leaves (Figures 6B,C). All these results suggested that the aroma quality of “Huangyu” came from hydrolyzing hydrolysis and carotenoid degradation in fresh leaves, and it was suitable for manufacturing non-fermented tea, e.g., green tea, white tea, or even yellow tea. While the typical aroma components of “Yinghong 9” came from fermentation processing, it was suitable for manufacturing fermented tea.

## Conclusion

In this study, analysis of non-volatiles and volatiles was conducted to identify the major metabolite compounds in “Yinghong 9” and its yellow-leaf mutant “Huangyu.” The contents of total carotenoids and chlorophyll revealed that chlorophyll concealed the coloration of carotenoids and led to the formation of normal green leaf in “Yinghong 9.” But for “Huangyu,” being the absence of chlorophyll in leaf, leaf coloration was predominated by carotenoids. The content of kaempferol-3- glucoside in “Yinghong 9” was 1.5 fold more than that of “Huangyu,” and the ratio of kaempferol-3-glucoside to kaempferol-3-galactoside was lower, indicating that “Huangyu” was suitable for manufacturing non-fermented tea, e.g., green tea and white tea, while “Yinghong 9” was suitable for manufacturing black tea. Analysis of carotenoid metabolites revealed that the contents of lutein and α-carotene were significantly higher in “Yinghong 9,” while zeaxanthin was the dominant compound in “Huangyu” leaves. In addition, the contents of nerolidol, α-farnesene and geranylacetone were significantly higher. And the higher activity of β-glucosidase and carotenoid cleavage dioxygenase in “Huangyu” fresh leaves also implied that “Huangyu” was suitable for manufacturing green tea. At the same time, nerolidol and geranyl acetone, the carotenoid degradation products, increased in “Yinghong 9” fermented leaves, which meant “Yinghong 9” was suitable for manufacturing black tea. Our current results provided a basis for understanding the mechanisms of yellow-leaf formation in “Huangyu” and facilitated the effective utilization for particular natural mutants.

## Data Availability Statement

The raw data supporting the conclusions of this article will be made available by the authors, without undue reservation.

## Author Contributions

XM designed the experiment. CL and XM carried out most of the experiments. LZ drafted the manuscript. BC and SW did the data analyses. LZ and HW revised and finalized the manuscript. All authors contributed to the article and approved the submitted version.

## Conflict of Interest

The authors declare that the research was conducted in the absence of any commercial or financial relationships that could be construed as a potential conflict of interest.

## Publisher’s Note

All claims expressed in this article are solely those of the authors and do not necessarily represent those of their affiliated organizations, or those of the publisher, the editors and the reviewers. Any product that may be evaluated in this article, or claim that may be made by its manufacturer, is not guaranteed or endorsed by the publisher.

## Footnotes

1
http://www.massbank.jp/

2
www.r-project.org

3
https://cloud.majorbio.com/

## Supplementary Material

Supplementary Table 1 Representative differential metabolites between “Yinghong 9” and “Huangyu” fresh leaves.

Supplementary Table 2 Differential metabolites between “Yinghong 9” fresh leaves and fermented leaves.

Supplementary Table 3 Differential metabolites between “Huangyu” fresh leaves and fermented leaves.

Supplementary Table 4 Representative flavonol glycosides between “Huangyu” fresh leaves and fermented leaves.

Supplementary Table 5A Differential flavonol glycosides between “Yinghong 9” fresh leaves and fermented leaves.

Supplementary Table 5B Representative flavonol glycosides between “Huangyu” fresh leaves and fermented leaves.

Supplementary Table 6 The quantification results of all detected carotenoids.

Supplementary Table 7 The results of volatile compounds in all samples.
